# Surgical (Open and laparoscopic) management of large difficult CBD stones after different sessions of endoscopic failure: A retrospective cohort study

**DOI:** 10.1016/j.amsu.2019.05.007

**Published:** 2019-05-31

**Authors:** Emad Hamdy Gad, Hazem Zakaria, Yasmin Kamel, Ayman Alsebaey, Talat Zakareya, Mohamed Abbasy, Anwar Mohamed, Ali Nada, Mohammed Alsayed Abdelsamee, Mohamed Housseni

**Affiliations:** a Hepatobiliary Surgery, National Liver Institute, Menoufia University, Shebein Elkoum, Egypt; b Anaesthesia, National Liver Institute, Menoufia University, Shebein Elkoum, Egypt; c Hepatology and Endoscopy, National Liver Institute, Menoufia University, Shebein Elkoum, Egypt; d Intervention Radiology, National Liver Institute, Menoufia University, Shebein Elkoum, Egypt

**Keywords:** Laparoscopic CBDE, Open CBDE, CBD stones

## Abstract

**Objectives:**

For complicated common bile duct stones (CBDS) that cannot be extracted by endoscopic retrograde cholangiopancreatography (ERCP), management can be safely by open or laparoscopic CBD exploration (CBDE). The study aimed to assess these surgical procedures after endoscopic failure.

**Methods:**

We analyzed 85 patients underwent surgical management of difficult CBDS after ERCP failure, in the period from 2013 to 2018.

**Results:**

Sixty-seven (78.8%) and 18(21.2%) of our patients underwent single and multiple ERCP sessions respectively. An impacted large stone was the most frequent cause of ERCP failure (60%). Laparoscopic CBDE(LCBDE), open CBDE(OCBDE) and the converted cases were 24.7% (n = 21), 70.6% (n = 60), and 4.7% (n = 4) respectively. Stone clearance rate post LCBDE and OCBDE reached 95.2% and 95% respectively, Eleven (12.9%) of our patients had postoperative complications without mortality. By comparing LCBDE and OCBDE; there was a significant association between the former and longer operative time. On comparing, T-tube and 1ry CBD closure in both OCBDE and LCBDE, there was significantly longer operative time, and post-operative hospital stays in the former. Furthermore, in OCBDE group, choledocoscopy had an independent direction to 1ry CBD repair and significant association with higher stone clearance rate, shorter operative time, and post-operative hospital stay.

**Conclusion:**

Large difficult CBDS can be managed either by open surgery or laparoscopically with acceptable comparable outcomes with no need for multiple ERCP sessions due to their related morbidities; furthermore, Open choledocoscopy has a good impact on stone clearance rate with direction towards doing primary repair that is better than T-tube regarding operative time and post-operative hospital stay.

## Highlights

• Large difficult CBD stones can be managed either by open surgery or laparoscopically with acceptable comparable outcomes.
• No need for multiple ERCP sessions due to their related morbidities.
• Open choledocoscopy has a good impact on stone clearance rate with direction towards doing primary repair.
• Primary repair is better than T-tube regarding operative time and post-operative hospital stay.

## List of abbreviations

ASA: American society of anesthesia
BDS: Bile duct stones
CBD: Common bile duct
CBDE: Common bile duct exploration
CBDS: Common bile duct stone
CHD: Common hepatic duct
CRP: C-reactive protein
EPBD: endoscopic papillary ballon dilatation
ES: Endoscopic sphincterotomy
ERCP: Endoscopic retrograde cholangio-pancreatography
HJ: Hepaticojejunostomy
HPB: Hepatopancreatobiliary
IOC: Intra-operative cholangiogram
IRB: Institutional review board
LC: Laparoscopic cholecystectomy
LCBDE: Laparoscopic common bile duct exploration
LCD: Laparoscopic choledochotomy
LFT: Liver function test
LTCE: Laparoscopic trans-cystic exploration
MRCP: Magnetic resonance cholangiopancreatography
NLI: National Liver Institute
OC: Open Cholecystectomy
OCD: Open choledochotomy
OCBDE: Open common bile duct exploration
PDS: polydioxanone
POD: Post-operative day
TDS: Transduodenal sphinectroplasty
US: Ultrasonography

## 1 Introduction

The incidence of common bile duct stones (CBDS) in patients with symptomatic cholelithiasis varies widely in the literature between 5% and 33% according to age [[1], [2], [3], [4], [5]]. CBDS are either primary (originating within the CBD) or secondary (originating in the gallbladder) and pass into the CBD [6,7]. Trans-abdominal ultrasound (US) and magnetic resonance cholangiopancreatography (MRCP) are the most common non-invasive pre-operative imaging modalities for detection of CBDS [8].

However, endoscopic retrograde cholangiopancreatography (ERCP) is the most common invasive tool for their detection. Treatment is advisable to prevent further complications, such as obstructive jaundice, acute cholangitis, and pancreatitis. [[8], [9], [10], [11]] Different modalities for successful treatment of these stones have been reported after advances in minimally invasive techniques as endoscopy, and laparoscopy, however, the optimal treatment is controversial [8,12,13]. They include one- or two-stage procedures; the two-stage procedures involve pre- or post- laparoscopic cholecystectomy- ERCP (LC-ERCP), while the single-stage procedures refer to Open or Laparoscopic CBD exploration (OCBDE or LCBDE) [2,10,[14], [15], [16]]. Pre- or postoperative ERCP is a popular treatment option commonly performed by endoscopists, nevertheless, it is associated with post-procedure complications [8,12,13]. Large, multiple and/or impacted stones in the CBD may be difficult or impossible to retrieve by ERCP [17,18]. Those patients can be managed with LCBDE or OCBDE, where these procedures have a high success rate in salvaging them [9,10,[18], [19], [20]]. To the best of our knowledge, there is little literature on surgical management of difficult CBD stones after ERCP failure, so, our study aimed to analyze this issue.

## 2 Patients and methods

One hundred patients underwent surgical management of large difficult CBDS after ERCP failure, in the period from the beginning of 2013 to the beginning of 2018 in the department of hepato-pancreato-biliary (HPB) surgery (tertiary care center), National Liver Institute (NLI), University of Menoufia, Menoufia, Egypt, our study included 85 patients after exclusion of cases with data loss, those who did not complete the follow-up and who refused researches. We did this cohort study which is a single-institution retrospective analysis of a prospectively collected database that assessed these surgical procedures of CBDE after endoscopic failure in the period from the beginning of 2013 to mid 2018, where patients were observed from POD1 until the end of June 2018 with a median follow up period of 39 ms, range (6–66 ms) [21]. The study was approved by Our IRB.

The data were collected from our records in our HPB surgery department and the endoscopic unit of hepatology department where written informed consents were obtained from patients regarding procedures, surgeries, and researches [21]. Our work has been reported in line with the STROCSS criteria [22], with researchregistry4588.

**Fig. 1:**
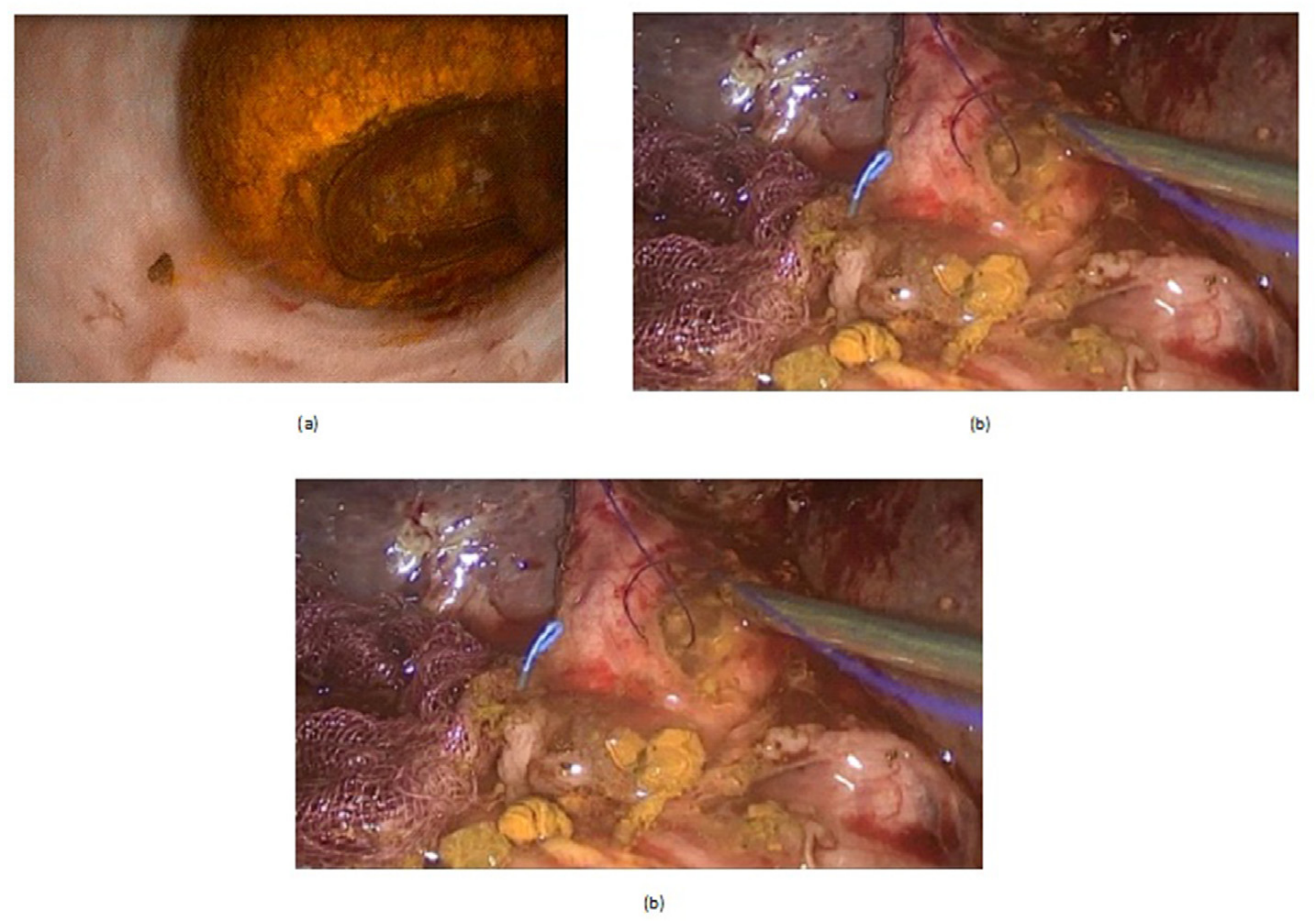
A: Laparoscopic cholecodoscopic view of CBD stone, B: laparoscopic cholecodoscopic stone extraction, C: laparoscopic primary closure of CBD.

**Fig. 2:**
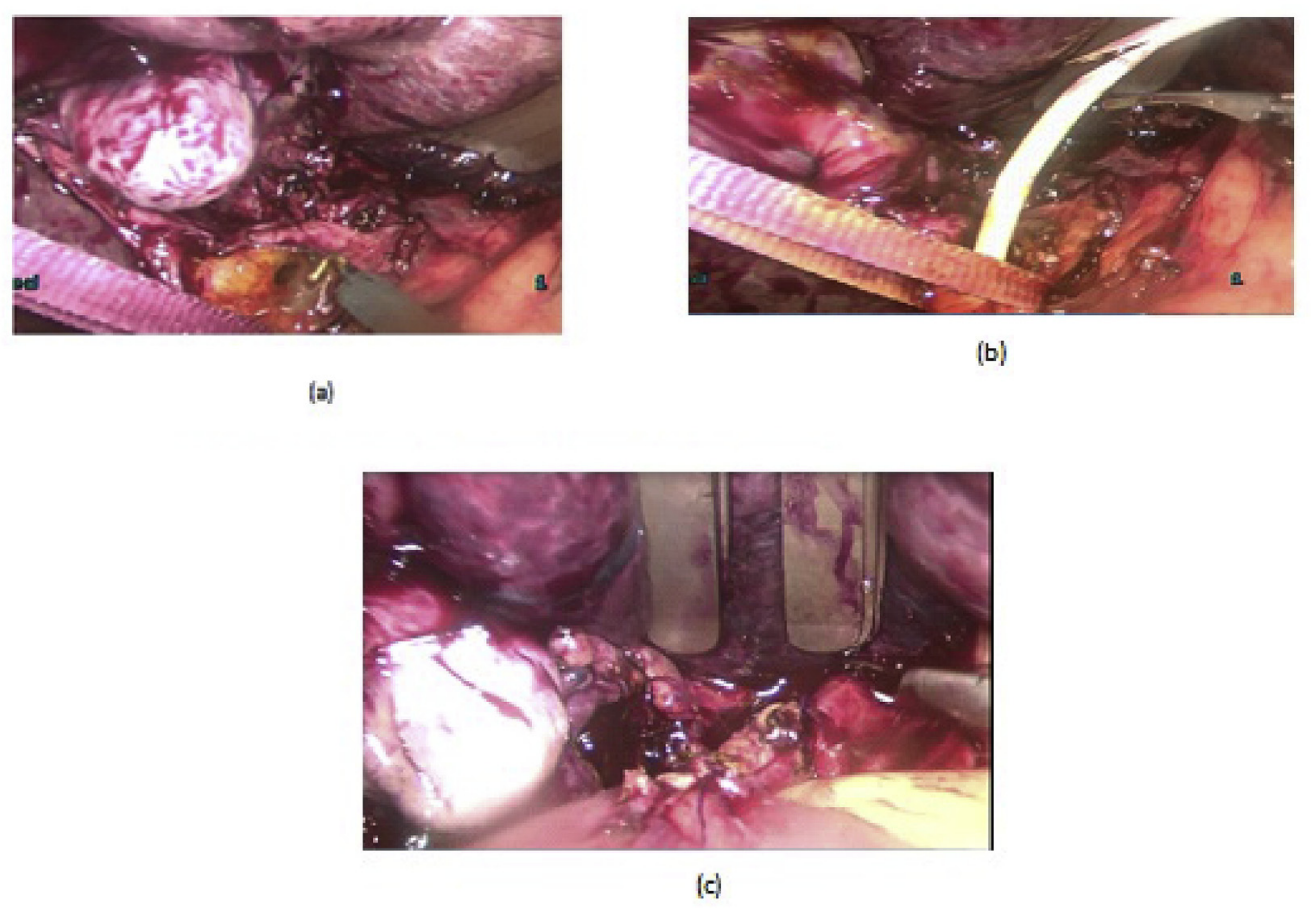
(a,b) laparoscopic stone and stent extraction, ^©^: laparoscopic HJ.

**Fig. 3:**
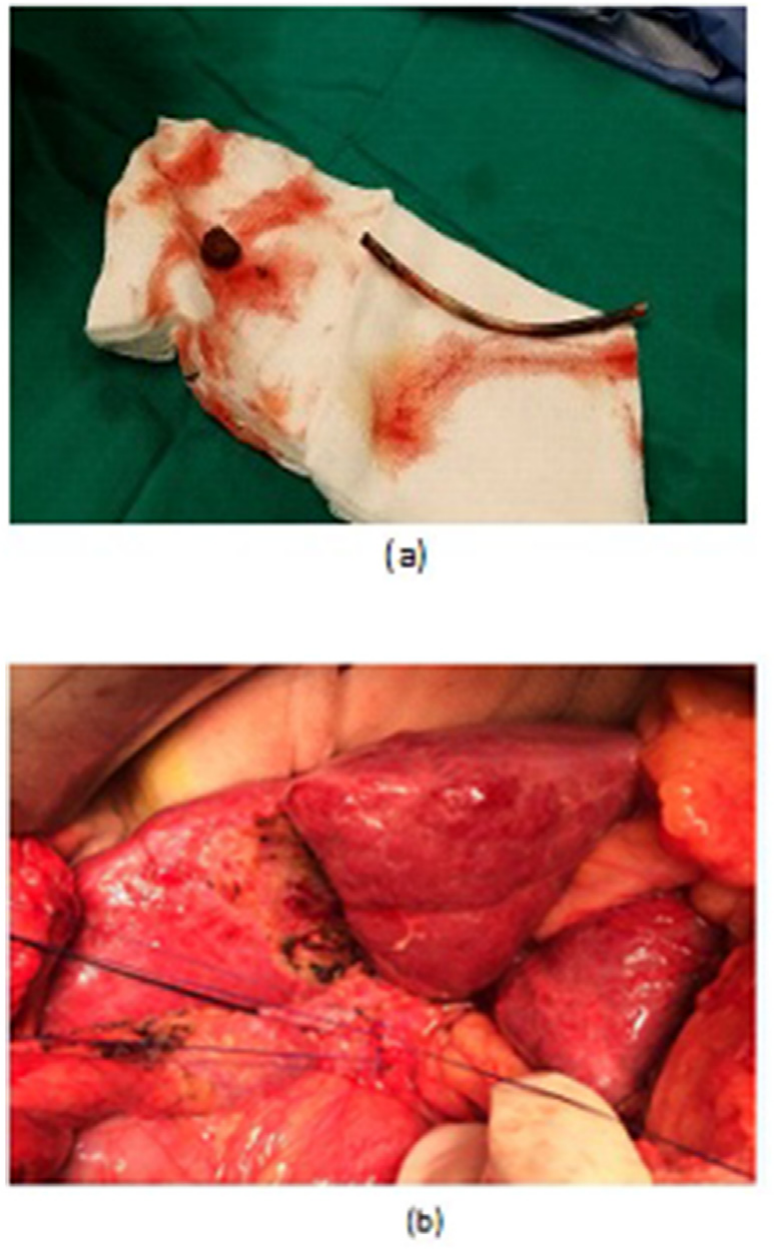
a: OCBD extraction of stent and stone. b: Open primary closure of CBD.

**Fig. 4:**
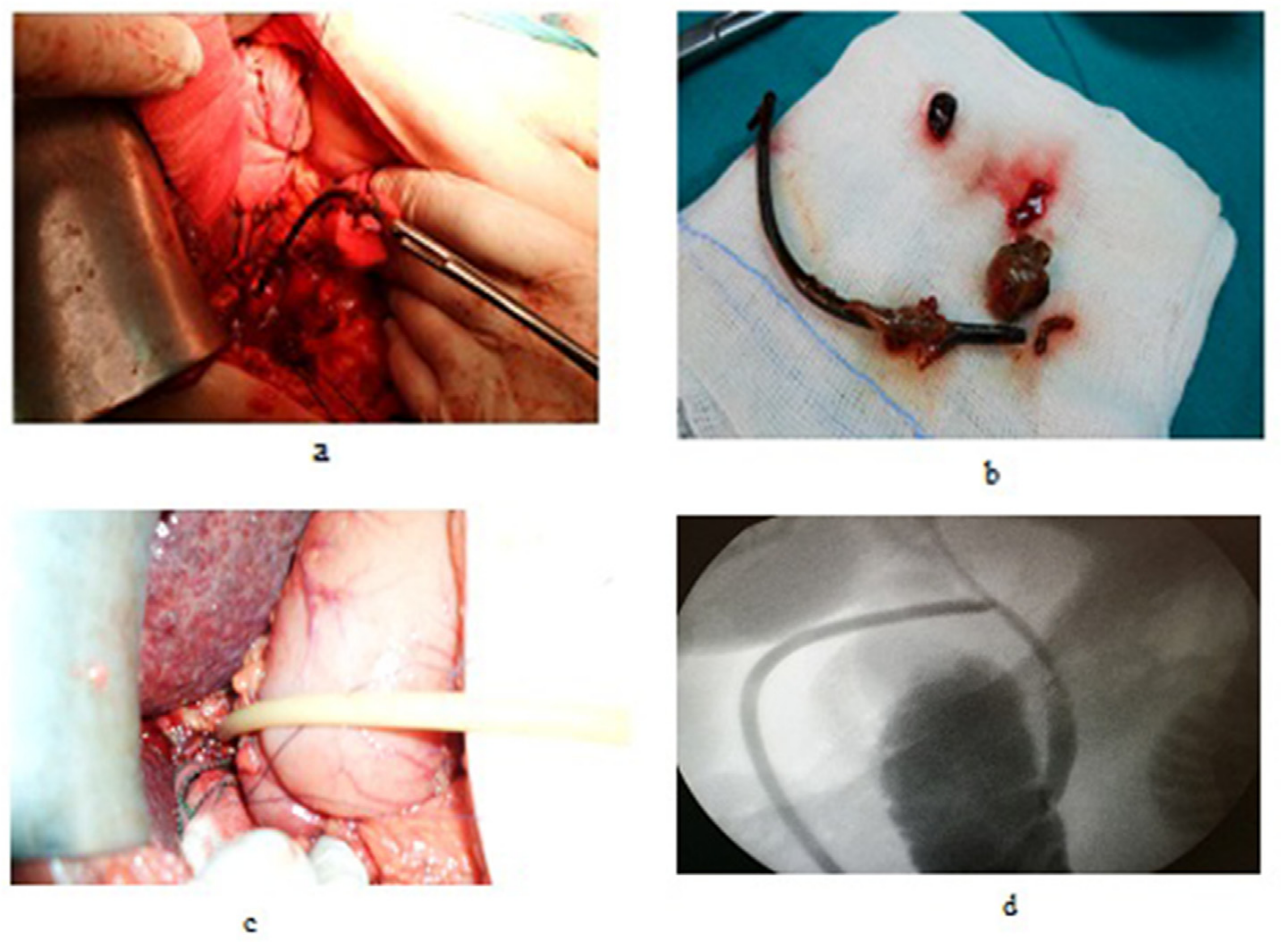
a, b, OCBD extraction of stent and stones. c,d: Open T-tube insertion, and T-tube cholangiogram.

**Fig. 5:**
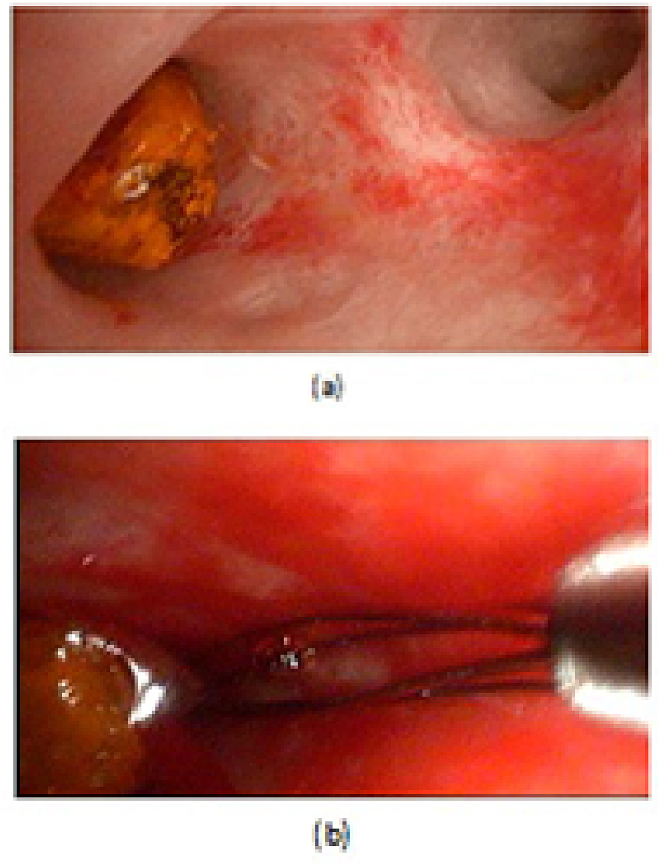
a: A cholecodoscopic view of stone in RT hepatic duct. b: Open cholecodoscopic basket extraction.

**Fig. 6:**
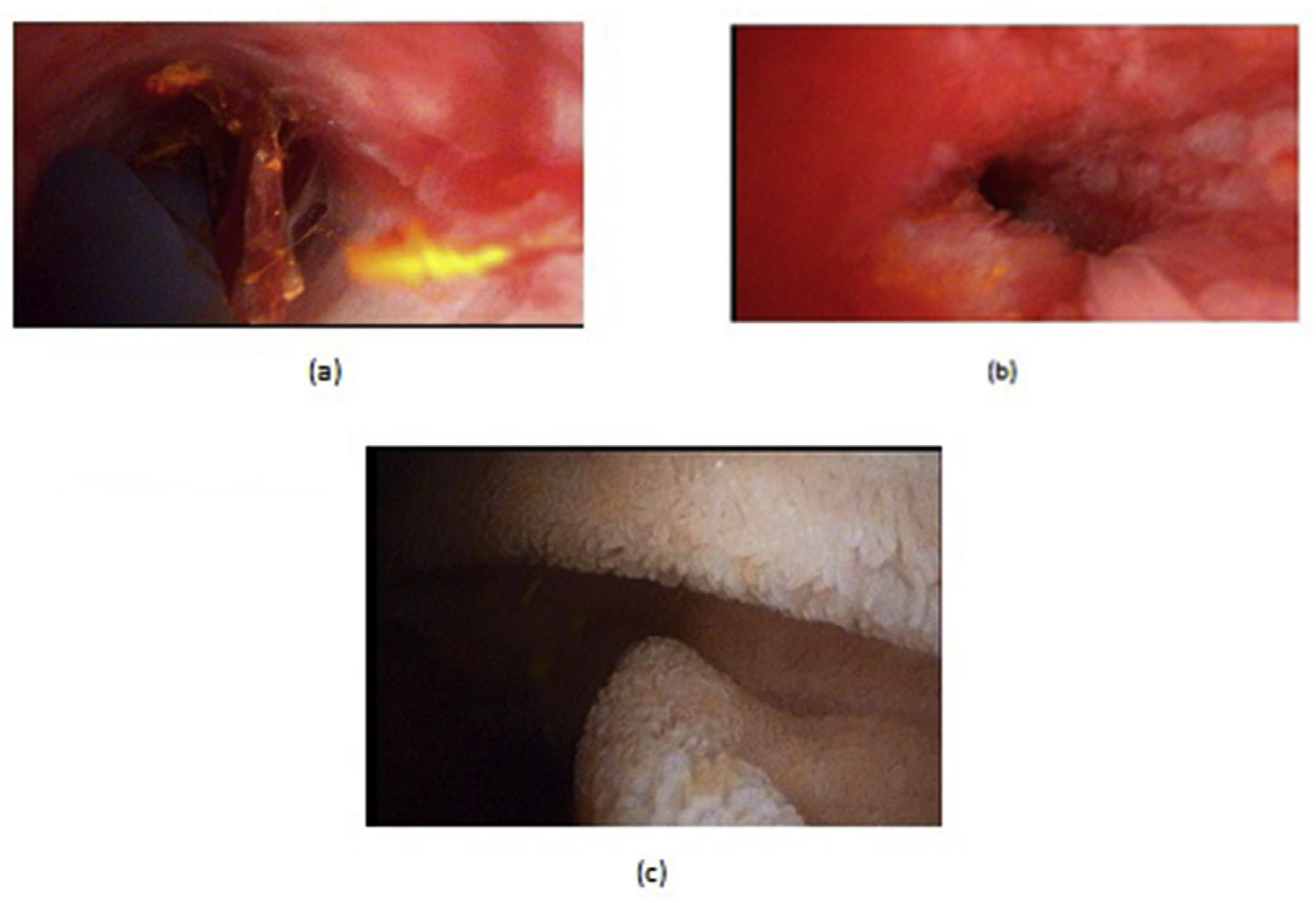
a: An open cholecodocoscopic view of stone and stent in distal CBD, b,c: cholecodoscopic extraction of stent and stone with duodenal mucosa appearance.

The recorded data included patient demographics, pre-ERCP main presentation, No of ERCP sessions, reasons of ERCP failure, post-ERCP complications, stone site ((ampullary, distal CBD, mid CBD, or common hepatic duct(CHD)), size(Small<1.5 cm, large1.5–2 cm, or very large< 2 cm), and NO(single or multiple), CBD diameter per mm, the pre-operative American society of anesthesia(ASA) score, operative details including: Type of operation: LCBDE(laparoscopic choledochotomy(LCD)), or OCBDE(supraduodenal open choledochotomy(OCD), or open transduodenal sphinectroplasty(TDS)), using cholecodoscope during surgery or not, causes of conversion from laparoscopic to open surgery, management of CBD after clearance from stones(primary repair, T-tube insertion, or hepaticojejunostomy(HJ)), operative bleeding, operative time per minutes, postoperative hospital stay per days, patient outcome and lastly follow-up data (Fig. 1, Fig. 2, Fig. 3, Fig. 4, Fig. 5, Fig. 6).

Pre-ERCP presentations (i.e. acute cholecystitis, biliary colic, jaundice, cholangitis, pancreatitis …) were confirmed by careful history taking, clinical examination, laboratory investigations especially liver function tests (LFTs), serum amylase, lipase and C-reactive protein (CRP), and by imaging as abdominal US ± MRCP.

The ERCP procedures were performed by the endoscopic authors of the manuscript with a side-viewing duodenoscope (JF-260 V or TJF-260 V; Olympus Medical Systems, Tokyo, Japan), under general anesthesia. Selective cannulation of the bile duct was achieved using a wire-guided sphincterotome (Clever Cut; Olympus Medical Systems). After successful cannulation, a contrast dye was injected to confirm the presence of CBDS that were extracted with the help of balloon or Dormia basket after performing endoscopic sphincterotomy(ES)(Mechanical lithotripsy was used for large stones (mechanical lithotripter BML 3Q and BML 4Q, Olympus, Tokyo, Japan). A check cholangiogram was performed to confirm the complete clearance of the bile duct. [15,[23], [24], [25]].

The patients were referred to our surgical department after single or multiple sessions of ERCP due to failure to cannulate CBD or to extract stones from CBD after successful cannulation due to their impaction and/or their large sizes despite using mechanical lithotripsy. All cases with successful cannulation but the failure of stone extraction were managed with single or multiple CBD stents (Cotton-Leung or Tannenbaum, Wilson-Cook, Winston-Salem, USA) with the 10-Fr diameter and 7–10 cm length put beside stones for drainage and possible stone fragmentation. The complications related to ERCP were recorded (I.e. bleeding, cholangitis, perforation, pancreatitis …).

### 2.1 The surgical techniques: (The operations were done by the surgical authors of the manuscript)

LCD: Under general anesthesia; we used the standard four-port technique of LC. Routine trans-cystic intra-operative cholangiography (IOC) was performed in all cases for identification of stones number, site and size. The gallbladder was left in situ for retraction until the operation was completed. When we reached CBD, a longitudinal supraduodenal choledochotomy(1.5–2 cm) was done using scissors or cautery. The CBD stones were entirely retrieved in all patients using cholecodoscopic extraction techniques (4.5-Fr flexible choledochoscope; Karl Storz, Tuttlingen, Germany) by irrigation, balloon or basket with mechanical lithotripsy when needed. The epigastric port was used to accommodate the operating choledochoscope [15,18,[26], [27], [28], [29]]. After all stones were retrieved and clearance of the bile duct was confirmed with choledochoscopy, the choledochotomy was closed with interrupted 4.0 Vicryl sutures in patients with primary closure of CBD and then IOC was done through the cystic duct to confirm absence of stones and leak (Fig. 1). On the other hand, for patients with T-tube drainage, the T-tube was placed in the choledochotomy and secured with sutures, Patients had a cholangiogram on the 6th postoperative day. If the finding was normal, the T-tube was clamped and patients were discharged home with the T-tube in situ then it was removed from 4 to 6 weeks later after normal tube cholangiogram in the outpatient clinic. [23,27,30]. Lastly, laparoscopic HJ was done by 4.0 polydioxanone (PDS) sutures (posterior and anterior interrupted sutures) and a tube drain was placed near the anastomosis and removed days after the operation (Fig. 2) [1,14,31,32].

OCD: It started by open cholecystectomy (OC) and IOC through the cystic duct; then identification of CBD and the junction between the cystic duct and the CBD were done. A complete Kocher's maneuver was performed in order to feel the retro- and intra-pancreatic portion of the CBD for easy extraction of stones and to feel the papilla. Then, two stay sutures were placed transversally at the right and left portions of the duct; then the anterior wall of the supraduodenal part of CBD was opened. The incision was performed with a sharp scalpel, then an exploration of the CBD first proximally and then distally with the Randall forceps occurred for extraction of visible stones. The proximal and distal CBD was then irrigated with saline using a soft catheter. In choledochoscopic cases, the CBD stones were entirely retrieved using choledochoscopic extraction techniques (4.5-Fr flexible choledochoscope; Karl Storz, Tuttlingen, Germany) (Fig. 5, Fig. 6). After all stones were extracted and clearance of the bile duct was confirmed; the management was as mentioned in LCD (Fig. 3, Fig. 4). Lastly, in HJ cases, the biliary-enteric anastomosis was done by 4.0 PDS sutures (posterior continuous and anterior interrupted sutures) [20].

TDS (for impacted stones in the ampulla of Vater): A Kocher maneuver was performed, after which a longitudinal anterior duodenotomy was made at the level of the ampulla, the ampulla and distal CBD were divided for a distance of 1.5–2 cm, directed anteromedially. The sphincter was divided sequentially between small clamps, with sequential suture approximation of the duodenal and bile duct mucosa using fine interrupted absorbable suture 4.0 vicryle. The duodenum was then closed transversely [8,33].

The outcome and follow-up of patients: For detection of postoperative complications (I.e. bile leak, missed, recurrent stones, and/or CBD stricture); patients were followed-up daily during hospital stay until discharge, then every 6 months in the 1st year then yearly until the end of follow-up period by clinical assessment, LFT, US, and others if needed (i.e. MRCP). Comparison between groups and subgroups of patients was done using univariate and multivariate analyses as follow: LCBDE vs. OCBDE, T-tube vs. 1ry repair in LCBDE group, T-tube vs. 1ry repair in OCBDE group, and choledochoscope vs. non-choledochoscope in OCBDE group.

Statistical Techniques: All data were processed with SPSS software (Statistical Product and Service Solutions, version 21, SSPS Inc, Chicago, IL, USA). Categorical variables were expressed in frequency and percentage and analyzed with the chi-square or Fisher exact tests. While continuous data were expressed as the mean and SD and were compared with the T or Mann whitteny tests. Univariate analysis and then multivariate analysis (by Binary logistic regression method) were done to compare different groups and subgroups of patients regarding pre-, intra-, and postoperative data. A P value of <0.05 was considered significant.

## 3 Results

### 3.1 Characteristics of patients regarding demographics and ERCP

They were classified as 27(31.8%) males, and 58 (68.2%) females; their mean age was 45.1 ± 11.5 years. Acute cholecystitis, biliary colic, cholangitis, jaundice, and pancreatitis were the main presentation pre-ERCP in 5(5.9%), 35 (41.2%), 5(5.9%), 36(42.4%), and 4(4.7%) of patients respectively. Sixty-seven (78.8%) of patients underwent single ERCP session, while 18 (21.2%) of them underwent multiple sessions. Very large, multiple large, impacted large stones and failed cannulation were the causes of ERCP failure in 13(15.3%), 17 (20%), 51(60%), and 4 (4.7%) of patients respectively. ERCP was complicated with bleeding, cholangitis, impacted dormia, and pancreatitis in 2.4%, 2.4%, 1.2%, and 3.5% of patients respectively. Table 1. On univariate analysis, there was a significant correlation between the number of ERCP sessions and post ERCP complications (27.8% complications with multiple sessions vs.4.5% with single ones; P = 0.009). Table 1.

**Table 1:** Characteristics of patients regarding demographics and ERCP.

Character	(Mean ± SD) or	
	No	(%)
Gender		
Males	27	(68.2%)
Females	58	(31.8%)
Age(years) (Mean ± SD)	45.1 ± 11.5	
Pre ERCP main presentation		
Acute cholecystitis	5	(5.9%)
Biliary colic	35	(41.2%)
Cholangitis	5	(5.9%)
Jaundice	36	(42.4%)
Pancreatitis	4	(4.7%)
No of ERCP sessions		
Single	67	(78.8%)
Multiple	18	(21.2%)
Cause of ERCP failure		
Very large stones(<2 cm)	13	(15.3%)
Multiple large stones	17	(20%)
Impacted large stones	51	(60%)
Failed cannulation	4	(4.7%)
Post ERCP complications	8	(9.4%)
Bleeding	2	(2.4%)
Cholangitis	2	(2.4%)
Impacted dormia	1	(1.2%)
Pancreatitis	3	(3.5%)

ERCP: Endoscopic retrograde cholangio-pancreatography.

Comparison between LCBDE, and OCBDE, and characteristics of the converted cases: The eighty-five patients were classified as follow: 21 patients completed LCBDE, 60 patients started and completed OCBDE, and 4 patients converted from LCBDE to OCBDE.

In LCBDE group (21 patients): They were classified as 6(28.6%) males, and 15 (71.4%) females; their mean age was 34.05 ± 6.1 years. Three (14.3%), 15(71.4%), and 3(14.3%) of patients had their stones in CHD, mid-CBD, and distal CBD respectively. The mean CBD diameter was 14 ± 4.7 mm, furthermore, the stones were classified into large (1.5–2 cm) and very large (>2 cm) in 18(85.7%) and 3(14.3%) of patients respectively. Nineteen (90.5%), and 2(9.5%) of patients had single, and multiple stones respectively. Stones were 1ry in 1 patient and 2ry in 20 ones. The preoperative ASA score was graded as I, II, and III in 14 (66.7%), 6 (28.6%), and 1(4.8%) of patients respectively. IOC and choledochoscopy were done for all patients. After stone extraction; Primary repair of CBD, T- tube insertion, and HJ were done in 9(42.9%), 11(52.4%), and 1 (4.8%) of them respectively. Operative bleeding affected 1 of patients. The mean operative times and hospital stays were 231.4 ± 49.3 min, and 5.5 ± 3 days respectively. Lastly, the success rate reached 95.2% Table 2. Regarding postoperative complications in this group, they affected 3(14.3%) of patients, where, chest infection, wound infection, missed stones, and bile leak complicated 1(4.8%), 1(4.8%), 1(4.8%), and 2(9.5%) of them respectively; patients with chest and/or wound infection were managed conservatively(grade II according to Clavien grading), patient with missed stone was managed successfully percutaneously with choledochoscopic CBDE through the biliary drainage sinus tract under fluoroscopic control(grade III), however, the 2 cases with biliary leak were managed successfully conservatively as the leak was minor(grade II). Lastly, there was no stricture, recurrent stones or mortality during the long-term follow-up. Table 2.

**Table 2:** Comparison between LCBDE, and OCBDE, and characteristics of the 4 converted cases.

Character	LCBDE (No = 21) (Mean ± SD) Or No (%)	OCBDE (No = 60) (Mean ± SD) or No (%)	P value Univariate analysis	P value Multivariate analysis	The converted cases (No = 4) (Mean ± SD) Or No (%)
Gender			>0.05		
Males	6(28.6%)	20 (33.3%)			1(25%)
Females	15(71.4%)	40 (66.7%)			3(75%)
Age(years)	34.05 ± 6.1	49.6 ± 9.9	0.000	0.05	36.3 ± 13.5
Site of stones			0.001	>0.05	
CHD	3(14.3%)	0			0
Mid-CBD	15(71.4%)	29(48.3%)			2(50%)
Distal CBD	3(14.3%)	26(43.3%)			2(50%)
Ampulla	0	5(8.3%)			0
CBD diameter(mm)	14 ± 4.7	15.4 ± 5.02	>0.05		14.3 ± 5.7
Stone size			0.1	>0.05	
Large(1.5–2 cm)	18(85.7%)	43(71.7%)			3(75%)
Very large(<2 cm)	3(14.3%)	17(28.3%)			1(25%)
NO of stones			>0.05		
Single	19(90.5%)	46(76.7%)			1(25%)
Multiple	2(9.5%)	14(23.3%)			3(75%)
Nature of stones			>0.05		
Primary	1(4.8%)	1(1.7%)			1(25%)
Secondary	20(95.2%)	59(98.3%)			3(75%)
ASA score			>0.05		
I	14 (66.7%)	40 (66.7%)			3(75%)
II	6 (28.6%)	16 (26.7%)			1(25%)
III	1(4.8%)	4(6.7%)			0
Type of operation					
LCD		0	0.000		4(100%)
OCD	0	55(91.7%)	0.000		4(100%)
TDS	0	5(8.3%)	0.1		0
IOC	21(100%)	60(100%)			4(100%)
choledochoscope	21(100%)	38(63.3%)	0.001	>0.05	4(100%)
Primary repair	9(42.9%)	30(50%)	>0.05		0
T- tube	11(52.4%)	20(33.3%)	0.1	>0.05	3(75%)
HJ	1(4.8%)	5(8.3%)	>0.05		1(25%)
Operative bleeding	1(4.8%)	2(3.3%)	>0.05		1(25%)
Operative time(min)	231.4 ± 49.3	160.7 ± 43.4	0.000	0.02	345 ± 13
Post-operative hospital stay(days)	5.5 ± 3	6.1 ± 2.1	>0.05		8 ± 1.4
Postoperative					
complications	3(14.3%)	7(11.7%)	>0.05	>0.05	1(25%)
Chest infection	1(4.8%)	5(8.3%)	>0.05		1(25%)
Wound infection	1(4.8%)	2(3.3%)	>0.05		0
Missed stones	1(4.8%)	3(5%)	>0.05		0
Bile leak	2(9.5%)	0	0.06		0
Stricture	0	0			
Recurrent stones	0	0			
Stone clearance rate	20 (95.2%)	57(95%)	>0.05		4(100%)
Mortality	0	0			0

LCBDE: Laparoscopic common bile duct exploration, OCBDE: Open common bile duct exploration, CHD: Common hepatic duct, CBD: Common bile duct, ERCP: Endoscopic retrograde cholangiopancreatography, ASA: American society of anaesthesia, LCD: Laparoscopic choledochotomy, OCD: Open choledochotomy, TDS: Transduodenal sphinectroplasty, IOC: Intra-operative cholangiogram, HJ: Hepaticojejunostomy.

In OCBDE group (60 patients): They were classified as 20(33.3%) males, and 40 (66.7%) females; their mean age was 49.6 ± 9.9 years. Twenty-nine (48.3%), 26(43.3%), and 5 (8.3%) of patients had their stones in mid-CBD, distal CBD, and ampulla of Vater respectively. The mean CBD diameter was 15.4 ± 5.02 mm, furthermore, the stones were classified into large (1.5–2 cm) and very large (>2 cm) in 43(71.7%) and 17(28.3%) of patients respectively. Forty-six (76.7%), and 14(23.3%) of patients had single, and multiple stones respectively. The mean time of referral to surgery after ERCP failure was 63.3 ± 86.6 days. Stones were 1ry in 1 patient and 2ry in 59 ones. The preoperative ASA score was graded as I, II, and III in 40 (66.7%), 16 (26.7%), and 4(6.7%) of patients respectively. OCD was performed in 55 patients, while TDS was the operation in 5 patients. IOC was done for all patients, while choledochoscope was done in 38(63.3%) of them. After stone extraction; Primary repair of CBD, T- tube insertion, and HJ were done in 30 (50%), 20(33.3%), and 5 (8.3%) of them respectively. Operative bleeding affected 2 of patients. The mean operative times and hospital stays were 160.7 ± 43.4 min, and 6.1 ± 2.1 days respectively. Lastly, success rate reached 95%. Table 2. The incidence of postoperative complications in this group was 7(11.7%) patients, where, chest infection, wound infection, and missed stones affected 5(8.3%), 2(3.3%), and 3(5%) of them respectively; patients with chest and/or wound infection (grade II Clavien) were managed conservatively, the 3 patients with missed stones (grade III) were managed successfully percutaneously with choledochoscopic CBDE through the biliary drainage sinus tract under fluoroscopic control. Lastly, there was no bile leak, stricture, recurrent stones or mortality during the long-term follow-up. Table 2.

When comparing both groups using univariate analysis; patient age was significantly lower, referral time was shorter, choledochoscope use was more frequent, and operative time was significantly longer in the laparoscopic group. On the other hand, on multivariate analysis, there was an independent correlation between longer operative time and LCBDE. Table 2.

Characteristics of the 4 patients converted from LCBDE to OCBDE: They were classified as 1(25%) males, and 3 (75%) females; their mean age was 36.3 ± 13.5 years. Two (50%), and 2(50%) of patients had their stones in mid and distal CBD respectively. The mean CBD diameter was 14.3 ± 5.7 mm (range; 10–22 mm), furthermore, the stones were classified into large (1.5–2 cm) and very large (>2 cm) in 3(75%) and 1(25%) of patients respectively. One(25%), and 3(75%) of patients had single, and multiple stones respectively. The mean time of referral to surgery after ERCP failure was 176.3 ± 18. 4 days (range; 15–300 days). The stones were classified as primary in 1 patient and secondary in 3 patients. The preoperative ASA score was graded as I, and II in 3(75%), 1(25%) of patients respectively. Intra-operative cholangiogram (IOC) and choledochoscopy were done for all patients. They were converted from LCBDE to OCBDE due to adhesions (1 patient), bleeding (1 patient), and technical failure (2 patients). After stone extraction; T- tube insertion, and HJ (1ry stone) were done in 3 (75%), and 1(25%) of them respectively. Operative bleeding affected 1 of them. The mean operative times and post-operative hospital stays were 345 ± 13 min, and 8 ± 1.4 days respectively. Lastly, postoperative chest infection affected 1 (25%) of them that was managed conservatively (Clavien II). Table 2.

Comparison between patients with T- tube insertion and primary closure of CBD:

1 In LCBDE group: On univariate analysis, there was a significantly longer operative time, and hospital stays in the subgroup of T-tube insertion. On the other hand, on multivariate analysis, there was no independent correlation between any variable and any of them. Table 3.

**Table 3:** T- tube insertion VS primary closure of CBD (LCBDE group).

Character	T- tube (No = 11) (Mean ± SD) or No (%)	Primary closure (No = 9) (Mean ± SD) or No (%)	P value Univariate analysis	P value Multivariate analysis
ASA score				
I	7(63.6%)	6(66.7%)	>0.05	
II	3(27.3%)	3(33.3%)		
III	1(9.1%)	0		
CBD diameter(mm)	13.3 ± 5.6	13.4 ± 0.9		
No of stones			0.1	<0.05
Single	9(81.8%)	9(100%)		
Multiple	2(18.2%)	0		
Operative time(min)	263.6 ± 37.8	191.1 ± 32.2	0.000	<0.05
Hospital stay(days)	7.6 ± 1	3 ± 2.6	0.001	<0.05
Postoperativ				
complications	2(18.2%)	1(11.1%)	>0.05	
Chest infection	1(9.1%)	0	>0.05	
Wound infection	1(9.1%)	0	>0.05	
Missed stones	1(9.1%)	0	>0.05	
Bile leak	1(9.1%)	1(11.1)	>0.05	
Stricture	0	0		
Recurrent stones	0	0		
Success rate	10(90.9%)	9(100%)	>0.05	

ASA: American society of anesthesia, CBD: Common bile duct.
2 In OCBDE group: On univariate analysis, there was a significant correlation between primary closure of CBD and smaller diameter of CBD, single stone, choledochoscopy, shorter operative times and hospital stays and between multiple CBD stones and T-tube insertion. However, on multivariate analysis, there was no independent correlation between any variable and T- tube insertion or primary CBD closure. Table 4.

**Table 4:** T- tube insertion vs. primary CBD closure in OCBDE group.

Character	T- tube (No = 20) (Mean ± SD)or No (%)	Primary closure (No = 30) (Mean ± SD) or No (%)	P value Univariate analysis	P value Multivariate analysis
ASA score				
I	15(75%)	21(70%)		
II	3(15%)	7(23.3%)		
III	2(10%)	2(6.7%)		
CBD diameter(mm)	19.7 ± 4.8	11.8 ± 2	0.000	>0.05
No of stones			0.000	>0.05
Single	8 (40%)	30(100%)		
Multiple	12(60%)	0		
choledochoscope	7(35%)	29(96.7%)	0.000	>0.05
Operative time(min)	177.5 ± 17.1	125.3 ± 15.9	0.000	>0.05
Hospital stay(days)	7.8 ± 0.8	4.4 ± 1.4	0.000	>0.05
Postoperative				
complications	3(15%)	3(10%)	>0.05	
Chest infection	2(10%)	3(10%)	>0.05	
Wound infection	1(5%)	0	>0.05	
Missed stones	3(15%)	0	0.05	>0.05
Bile leak	0	0		
Stricture	0	0		
Recurrent stones	0	0		
Success rate	17(85%)	30(100%)	0.05	>0.05

ASA: American society of anesthesia, CBD: Common bile duct.

Comparison between patients with and without choledochoscope usage in OCBDE group: On univariate analysis, there was a significant correlation between intra-operative choledochoscope and the followings: Primary CBD repair, shorter operative time, shorter hospital stay, lower missed stones, and higher stone clearance rates. However, on multivariate analysis, there was an independent correlation between choledochoscope and performing primary repair of CBD after stone extraction. Table 5.

**Table 5:** Choledochoscope usage in OCBDE group.

Character	Choledochoscope (No = 38) (Mean ± SD) or No (%)	No choledochoscope (No = 22) (Mean ± SD) or No (%)	P value Univariate analysis	P value Multivariate analysis
ASA score				
I	25(65.8%)	15(68.2%)	>0.05	
II	10(26.3%)	6(27.3%)		
III	3(7.9%)	1(4.5%)		
Primary repair	29(76.3%)	1(4.5%)	0.000	0.02
T- tube	7(18.4%)	13(59.1%)	0.002	<0.05
Operative bleeding	2(5.3%)	0	<0.05	<0.05
Operative time(min)	140.8 ± 36.1	195 ± 32.3	0.000	<0.05
Hospital stay(days)	5.3 ± 2	7.3 ± 1.4	0.000	0.06
Postoperative				
complications	4(10.5%)	3(13.6%)	<0.05	
Chest infection	3(7.9%)	2(9.1%)	<0.05	
Wound infection	1(2.6%)	1(4.5%)	<0.05	
Missed stones	0	3(13.6%)	0.04	<0.05
Bile leak	0	0		
Stricture	0	0		
Recurrent stones	0	0		
Stone clearance rate	38(100%)	19(86.4%)	0.04	<0.05

ASA: American society of anesthesia.

## 4 Discussion

Various options for managing CBDS are available such as ERCP, LCBDE, and OCBDE [33,34]. However, ERCP followed by LC for managing concomitant gallbladder and CBDS is currently the preferred method in the majority of hospitals worldwide [2]. Similarly, it is the preferred method in our institute.

ERCP failure to extract stones may be due to failed cannulation (i.e., Juxta-papillary diverticulum, intra-diverticular papilla or small papilla), or failed extraction [[35], [36], [37], [38], [39]]. The failed extraction occur with difficult stones (i.e Mirrizi's syndrome, stricture of the lower CBD, impacted, large (<15 mm), multiple (<3), or intrahepatic duct/cystic duct stones), especially when using standard methods (balloon or basket after ES or endoscopic papillary balloon dilatation (EPBD)) [39,40]. In our study, failed cannulation, very large (<2 cm), multiple large, and/or impacted large stones were the causes of ERCP failure. On the other hand, in Bansal et al., 2014 [23] study, the failure was due to the inability to identify the papilla, unsuccessful cannulation, impacted stones, and duodenal perforation. However, previous operations, anatomic abnormalities and stone impaction were the causes of ERCP failure in Karaliotas et al., 2008 [41] study.

When CBDS clearance become unsuccessful, temporary stenting can serve as a bridge preventing stone impaction and cholangitis by relieving biliary obstruction and ensuring biliary drainage for further planned endoscopic stone removal or operation [3,36]. Furthermore, biliary stenting has some therapeutic benefit in case of difficult stones (I.e. difficult stones become smaller, fragmented and easier to remove at repeat ERCP or even absent after a period of stenting) [25,42,43]. Similarly, in our work, all cases with failure of stone extraction after successful cannulation were managed with single or multiple CBD stents put beside stones for drainage and possible stone fragmentation till further planned endoscopic or surgical extraction.

Post ERCP complications vary widely in the literature between 5 and 38%; due to pancreatitis, duodenal perforation, bleeding, cholangitis, and papillary stenosis [13,18,32,[44], [45], [46], [47], [48], [49], [50], [51]] It was 9.4%, 11%, and 11.1% in ours, Tai et al., 2004 [19], and Koc et al., 2013 [34] studies respectively. Where our ERCP was complicated with pancreatitis, bleeding, cholangitis, and impacted dormia in 3.5%, 2.4%, 2.4%, and 1.2% of patients respectively.

After ERCP failure, the treatment options are either LCBDE or OCBDE [45,52,53]. Furthermore, they can be performed in the complex [36], and recurrent CBDS [54], because repeated ERCP has increased complication rate [55]. Similarly, in our work, there was a significant correlation between the number of ERCP sessions and post ERCP complications.

With the advancement in laparoscopic techniques, development of new instruments for CBDE and increased experience in laparoscopic biliary surgery, many centers have started performing LCBDE with acceptable results as it is safe and efficient in the treatment of BDS [23,34,45]. Moreover, it requires training, standardization of surgical technique, accurate positioning of the trocars, a certain degree of expertise, and specific operative equipment [27,56]. Furthermore, It became the gold standard for CBDS removal when ERCP fails, [32,57,58] It can be performed by the trans-cystic approach (LTCE) or by LCD [56]. The choice of the approach is made according to the number, size, location of stones, Cystic duct, and CBD diameters, and anatomy of the cystic duct-CBD junction [4,13,14,56,59,60]. LCD is used in case of difficult, impacted, large, and/or multiple stones, and failed LTCE [15,19,35]. Moreover, it reduces the need for second invasive ERCP with reduction of costs and patient discomfort [61]. Furthermore, It provides unrestricted visualization of the biliary system, allows retrieval of difficult stones located in the extra-hepatic or intra-hepatic biliary tree, and carries a higher clearance rate than the trans-cystic approach. [1,29,32]Similarly, we started performing LCBDE recently, where Our LCBDE after ERCP failure was through LCD. In the same line, after ERCP failure, most LCBDE procedures (96%) were performed via choledochotomy in Tang and Li, 2005 [26] study, and 27.7% of LCDs were referred from the gastroenterology unit after one or more failed trials at endoscopic clearance of difficult stones in Paganini et al., 2005 [61] study.

LCD stone clearance rate ranges from 58.3% to 100%, [8,46,62] similarly, it was 80% in our study when including converted cases and 95.2% if excluding them. However, LCD stone clearance rate after ERCP failure was 62.5%, 64.51%, and 100% in Karaliotas et al., 2015 [28], Karaliotas et al., 2008 [41], and Karunadasa et al., 2016 [63] studies respectively, and it was 95.65% when performed after ERCP failure in non-dilated CBD in Jinfeng et al., 2016 [32] study, while it was 83.3% for impacted stones in Khan et al., 2015 [64] study. On the other hand, LCD stone clearance rate ranged between 82% and 100% in Mattila et al., 2017 [65], Quaresima et al., 2017 [4] Tokumura et al., 2002 [66], Grubnik et al., 2012 [67], Jinfeng et al., 2016 [1], Jinfeng et al., 2016 [32], Khaled et al., 2013 [68], Zhou et al., 2017 [15], Zhan et al., 2016 [7], and Vindal et al., 2015 [69] studies.

After LCD, the morbidity rate ranges from 4% to 26.7%, [8,46,62] similarly, after ERCP failure, LCD complication rate was 12.5%, 14.3% and 18.8% in Karaliotas et al., 2008 [41], ours and Karaliotas et al., 2015 [28] studies respectively, however, after ERCP failure, LCD in non-dilated CBD had 8.7% complication rate in Jinfeng et al., 2016 [32] study, on the other hand, the morbidity rate post-LCD ranged between 8.3% and 26.6% in Khaled et al., 2013 [68], Mattila et al., 2017 [65], Quaresima et al., 2017 [4], and Deo et al., 2018 [70] studies. Conversely, there was no major complication after LCD in Zhan et al., 2016 [7] study.

After ERCP failure, post LCD, missed stone rate was 1.09%, 3.2%, and 4.8% in Karaliotas et al., 2008 [41], Karaliotas et al., 2015 [28], and ours studies respectively, however, after ERCP failure, LCD in non-dilated CBD had no retained stone in Jinfeng et al., 2016 [32] study, while the retained stone rate after LCD for impacted stone reached 6.7% in Khan et al., 2015 [64] study, on the other hand, the retained stone rate after LCD was in the range of 1.2% and 8% in Jinfeng et al., 2016 [32], Jinfeng et al., 2016 [1], Mattila et al., 2017 [65], Khaled et al., 2013 [68], Grubnik et al., 2012 [67], Quaresima et al., 2017 [4], Tokumura et al., 2002 [66], and Paganini et al., 2005 [61] studies, conversely, there was no retained stone rate after LCD in Zhan et al., 2016 [7], and Vindal et al., 2015 [69] studies.

The LCD performed after ERCP failure had 6.3%, and 9.5% bile leaks in Karaliotas et al., 2015 [28], and our studies respectively, while LCD done in non-dilated CBD after ERCP failure had 4.35% bile leak in Jinfeng et al., 2016 [32] study, on the other hand, post LCD bile leak was in the range of 1.6% and 11% in Jinfeng et al., 2016 [1], Quaresima et al., 2017 [4], Paganini et al., 2005 [61], Jinfeng et al., 2016 [32], Khaled et al., 2013 [68] Vindal et al., 2015 [69], Tokumura et al., 2002 [66], Zhou et al., 2017 [15], Mattila et al., 2017 [65], and Reinders et al., 2014 [46] studies.

On long-term follow-up after LCD, we did not detect any recurrent stones, similarly, Khaled et al., 2013 [68] had no recurrent stone after their LCD, however, after ERCP failure, LCD in non-dilated CBD had 4.35% recurrent stone rate in Jinfeng et al., 2016 [32] study, on the other hand, the incidence of recurrent stone after LCD ranged between 1.3% and 4.3% in Jinfeng et al., 2016 [1], Jinfeng et al., 2016 [32], Tokumura et al., 2002 [66], Quaresima et al., 2017 [4] and Zhou et al., 2017 [15] studies.

There was no biliary stricture after our LCD, in similar, Jinfeng et al., 2016 [32] did not detect stricture after their LCD that was performed in non-dilated CBD after ERCP failure, also, there was no stricture post LCD in Jinfeng et al., 2016 [1], Quaresima et al., 2017 [4], Cai et al., 2012 [30], Jinfeng et al., 2016 [32], and Paganini et al., 2005 [61] studies. In contrast, Khaled et al., 2013 [68] found 0.8% post LCD stricture.

Despite advances in LCD, previous upper abdominal operations, dense adhesions, impacted, multiple stones, bleeding, and technical difficulties are causes of conversion to open surgery [1,66,70,71]. In similar, There were 4 cases of conversion in our series due to adhesions (1 patient), bleeding (1 patient), and technical failure (2 patients); this conversion reached 16%(4/25), however, the LCD done after ERCP failure conversion rate was 34.3% and 34.4% in Karaliotas et al., 2015 [28], and Karaliotas et al., 2008 [41] studies respectively, while it reached 4.35% in Jinfeng et al., 2016 [32] study that was performed in non-dilated CBD after endoscopic failure, and reached 10% in Khan et al., 2015 [64] study for impacted stones, on the other hand, LCD conversion to open ranged between 0.8% and 26.6% in Tokumura et al., 2002 [66], Grubnik et al., 2012 [67], Jinfeng et al., 2016 [32]. Jinfeng et al., 2016 [1], Quaresima et al., 2017 [4], Korontzi et al., 2012 [18], Mattila et al., 2017 [65], and Deo et al., 2018 [70] studies. Conversely, there was no post-LCD conversion in Zhan et al., 2016 [7], Khaled et al., 2013 [68], and Vindal et al., 2015 [69] studies.

Despite development in endoscopic and laparoscopic techniques, OCBDE is still the choice in some hospitals in developing countries [20,58], in many surgical clinics [66], in eastern Europe [67], in many Asian countries, [27] and in some patients ((I.e. previous surgery with dense adhesions, aberrant biliary ductal anatomy, ……) [8,72]. Furthermore, it is indicated after failure of ERCP [18,26,33,41,73,74]. Similarly, after failure of ERCP, OCBDE was the main procedure in our institution (60/81; 74.1% if excluding converted cases, and 64/85; 75.3% if including them); the reason for this is that OCBDE was our usual operation as we started LCBDE very recently.

The success rate of OCBDE ranges from 89% to 97% [17]. Similarly, it reached 98% in our study. On the other hand, the complication rate after those procedures may reach 11–14% [45]. However, it was 8%, 11.7% and 19% in Neoptolemos et al., 1987, [75] ours, and Kapoor et al., 1996 [76] studies respectively. In another line, the OCBDE performed after ERCP failure had 27% complication rate in Jalal et al., 2018 [72] study.

The retained stone rate after OCBDE ranges from 1% to 8% [73,77]. Similarly, it was 5% in our series, however, Şahiner and Kendirci, 2017 [53] did not detect any retained stones after their OCBDE. On the other hand, after long-term follow-up, we did not detect any bile leak, recurrent stones or biliary strictures after our OCBDE, similarly, Şahiner and Kendirci, 2017 [53] did not find any recurrent stones after their OCBDE. However, Jalal et al., 2018 [72] found 18% bile leak after their OCBDE that was done after ERCP failure, while, Escarce et al., 1995 [78] detected 1.1% strictures after their OCBDE that was performed in the pre-laparoscopic era.

LCBDE when performed by an experienced surgeon results in no additional morbidities as compared to OCBDE, with excellent success rates thus benefits patients with multiple, large and/or impacted stones in a dilated CBD [27]. Similarly, in our work, there was no difference between both techniques regarding morbidities or stone clearance rate with an excellent success rate in both procedures (≤95%). Also, Lin et al., 2017 [79] did not find a difference between both techniques regarding morbidity or stone clearance. In contrast, LCBDE had less morbidity when compared to OCBDE in Qiu et al., 2015, [58] and Grubnik et al., 2012 [76] studies.

We did not find any difference between LCBDE and OCBDE regarding intra-operative bleeding or post-operative hospital stays, conversely, Qiu et al., 2015, [58] and Grubnik et al., 2012 [76] found less bleeding and shorter hospital stay with the LCBDE technique. Similarly, Shelat et al., 2015, [71] and Lin et al., 2017 [79] detected shorter hospitalization with LCBDE. However, on comparing LCBDE with open surgery, we found significant independent longer operative time in the former, similarly, Lin et al., 2017 [79] found significant longer operative time with the LCBDE procedure, on the other hand, there was no difference between both procedures regarding operative time in Shelat et al., 2015, [71] or Grubnik et al., 2012 [76] studies.

After stone removal during LCD, the ductotomy is usually closed either over a T-tube or primarily [80]. Despite, T-tubes are used to prevent bile stasis, decompress the biliary tree by decreasing intra-biliary pressure and edema, achieve a controlled biliary fistula, and provide a percutaneous access for extraction of missed stones; [10,13,81] they have related complications (I.e. Tube displacement, bile leakage, peritonitis, persistent biliary fistula, cholangitis, prolonged hospital stay, fluid and electrolyte disturbances, sepsis, localised pain, discomfort and late biliary stricture, [30,53,68,81] So, primary closure of the CBD after LCD is a safe and efficient alternative with excellent results, avoiding T-tube related complications [56,59]. It can be done when complete stone extraction is ensured during the operation especially with choledochoscopy [66,82]. We used T-tube in 52.4% of our LCD patients; similarly, it was used in the range of 32.3% and 86.7% of patients in Herrero et al., 2013, [56] Tokumura et al., 2002, [66] Grubnik et al., 2012, [67] Quaresima et al.,2017, [4] Paganini et al., 2005, [61] Karaliotas et al., 2008, [41] Mattila et al., 2017, [65] and Deo et al., 2018 [70] studies. On the other hand, primary closure of CBD was performed in 42.9% of our LCD patients, in similar; its use ranged from 5.1% to 100% of patients in Paganini et al., 2005 [61], Tokumura et al., 2002 [66], Quaresima et al., 2017 [4], Grubnik et al., 2012 [67], Herrero et al., 2013 [56], and Koc et al., 2013 [34] studies.

On comparing primary closure with T-tube after LCD, we found a significantly shorter operative time and post-operative hospital stay in the former; similarly, there was shorter operative time and post-operative hospital stay with primary closure technique in different literature studies. Wu et al., 2012 [11], Podda et al., 2016 [13], Zhang et al., 2015 [14], Cai et al., 2012 [30], Herrero et al., 2013 [56], and Khaled et al., 2013 [68] However, T-tube was associated with longer postoperative stay and the time until return to work in Leida et al., 2008 [81] study.

In our study, after LCD, the T-tube was associated with a higher complication rate in comparison to primary closure (18.2% vs 11.1%). Also, Herrero et al., 2013 [56], Khaled et al., 2013 [68], Zhang et al., 2015 [14], Leida et al., 2008 [81], and Wu et al., 2012 [11] noticed similar findings. However, we found less biliary complication rate on comparing primary closure with T-tube after LCD (11.1% vs 18.2% respectively), similarly, Leida et al., 2008 [81] found less biliary complication in the primary closure group, also, Wu et al., 2012 [11] found less biliary complication without a combination of retained stone in the primary closure group, and Podda et al., 2016 [13] found that primary closure was associated with less biliary peritonitis.

After LCD, Tokumura et al., 2002 [66] found a higher incidence of bile leak and lower incidence of retained stone in their primary closure subgroup of patients. In similar, our primary closure technique had a higher biliary leak and lower missed stone in comparison to T-tube (11.1% vs 9.1%, and 0 vs 9.1% respectively). However, Leida et al., 2008 [81] detected a similar incidence of bile leak and missed stone rate between both subgroups of patients (5%, and 2.5% respectively). On the other hand, Cai et al., 2012 [30] noticed comparable bile leak with no missed stone in any of the subgroups. However, on long-term follow-up they did not detect any recurrent stones or CBD stricture, either T-tube or primary closure were done. Similarly, we did not find any recurrence or stricture after any of the techniques.

T-tube placement after OCBDE for stones was the traditional tool for decompression of the CBD and extraction of residual stones through the T-tube tract [11,13,67]. However, T-tube after OCBDE had its related complications (I.e. Wound infection, bile leakage, persistent biliary fistula, cholangitis, prolonged hospital stay, and CBD stenosis) [10,53,[83], [84], [85]]. So, primary closure of CBD after OCD is supported by some authors Seale and Ledet, 1999 [86]. It is safely done with a normal duct when the surgeon is satisfied with CBD clearance [87] and has experienced hands [20]. In similar, we performed primary closure after OCD when we were satisfied with CBD clearance by choledochoscope and by stone number where there was a significant correlation between our 1ry closure and both choledochoscope and single stones.

On comparing primary closure with T-tube drainage during OCD, we found a significant correlation between the former and shorter operative times and hospital stays with comparable complication rates. Also, Williams et al., 1994 [88] detected similar findings, while, Yamazaki et al., 2006 [89] found a significant reduction in postoperative hospital stay with primary closure when compared with T-tube with comparable complication rate, however, primary closure of CBD after OCD leads to a significantly less hospital stay in comparison to T-tube in Ambreen et al., 2009(85) study.

Choledochoscope that can be introduced through the cystic duct or CBD enables direct visualization of both extra-hepatic and intra-hepatic biliary systems ensuring their complete stones clearance with inspection of the distal bile duct for sphincter of Oddi abnormalities or retained stones [1,7,18]. It can be used with difficult stones [90]. Furthermore, it has various advantages over IOC: Better view, absence of fluoroscopy, lithotripsy performance under direct vision, a lower rate of T-tube usage, shorter operating time, higher rates of CBD clearance, and low rate of missed stones [1,7,18,27,35,85].

We used choledochoscopy in all our LCD cases with 95.2% stone clearance rate and 4.8% missed stone rate. Similarly, Koc et al., 2013 [34], and Chander et al., 2011 [27] used choledochoscope in all their LCBDE cases to ensure stone clearance. In the same line, LCD stone clearance rate using choledochoscopy reached 95%, 96.5% and 100% in Korontzi et al., 2012 [18], Karaliotas et al., 2015 [28] and Tekin, and Ogetman, 2010 [35] studies respectively. While, the retained stone rate after laparoscopic choledochoscopy ranged from 0 to 3.5% in Cai et al., 2012 [30]. Chander et al., 2011 [27] and Karaliotas et al., 2015 [28] studies.

We found a significant correlation between our open choledochoscopy and both higher stone clearance rate and lower missed stone rates, similarly, Ford et al., 2011 [91], Desai and Shokouhi, 2009 [87], and Korontzi et al., 2012 [18] detected better stone clearance rate when open choledochoscopy was used where these rates ranged between 97% and 98% in their studies. On the other hand, Takada et al., 1991 [92], and Schwarz et al., 2007 [93] found lower missed stone rate with open choledochoscopy.

To the best of our knowledge, the literature did not discuss the correlation between open choledochoscopy and performing primary CBD closure after stone removal, however, we found an independent correlation between both issues, with associated shorter operative times and hospital stays, the explanation is that when we used choledochoscope, we were satisfied with CBD stone clearance, so primary closure was done without fear of missed stones, with associated shorter both operative times and post-operative hospital stays. In conclusion: Large difficult CBD stones can be managed either by open surgery or laparoscopically with acceptable comparable outcomes with no need for multiple ERCP sessions due to their related morbidities; furthermore, Open choledochoscopy has a good impact on stone clearance rate with direction towards doing primary repair that is better than T-tube regarding operative time and hospital stay.

## Financial support

Forms of support received by each author for this study included good selection of cases, instructive supervision, continuous guidance, valuable suggestions, and good instructions. Furthermore, the authors of the manuscript shared in its data collection, writing, and publication; moreover, the corresponding author did statistical analysis as well.

## Ethical approval

The approval by National liver institute (IRB), Menoufia university that was done retrospectively as it was not working at the time of study.

## Sources of funding

No source of funding for this research.

## Author contribution

Emad Hamdy Gad: Study design, data collection, writing, statistical analysis and publication.

Hazem Zakaria: data collection, writing.

Yasmin Kamel: data collection, writing, statistical analysis.

Ayman Alsebaey: data collection, writing.

Talat Zakaria: data collection, writing.

Mohamed Abbasy: data collection, writing.

Anwar Mohamed: data collection, writing.

Ali Nada: data collection, writing.

Mohamed Housseni: data collection, writing.

Mohammed Al-sayed Abd-elsamee: Reference update.

## Conflicts of interest

No conflict of interest to declare.

## Trial registry number

Researchregistry4588.

## Guarantor

All the authors of this paper accept full responsibility for the work and/or the conduct of the study, had access to the data, and controlled the decision to publish.

## Provenance and peer review

Not commissioned, externally peer reviewed.
